# Evolution of biomedical ontologies and mappings: Overview of recent approaches

**DOI:** 10.1016/j.csbj.2016.08.002

**Published:** 2016-08-26

**Authors:** Anika Groß, Cédric Pruski, Erhard Rahm

**Affiliations:** a Institute of Computer Science, Universität Leipzig, P.O. Box 100920, 04009 Leipzig, Germany; b Luxembourg Institute of Science and Technology, 5 Avenue des Hauts-Fourneaux, L-4362 Esch-sur-Alzette, Luxembourg

**Keywords:** Biomedical ontology, Ontology evolution, Ontology-based mapping, Mapping evolution, Mapping adaptation, Biomedical annotation

## Abstract

Biomedical ontologies are heavily used to annotate data, and different ontologies are often interlinked by ontology mappings. These ontology-based mappings and annotations are used in many applications and analysis tasks. Since biomedical ontologies are continuously updated dependent artifacts can become outdated and need to undergo evolution as well. Hence there is a need for largely automated approaches to keep ontology-based mappings up-to-date in the presence of evolving ontologies. In this article, we survey current approaches and novel directions in the context of ontology and mapping evolution. We will discuss requirements for mapping adaptation and provide a comprehensive overview on existing approaches. We will further identify open challenges and outline ideas for future developments.

## 1 Introduction

Ontologies have gained much importance in the past two decades, especially in the biomedical domain [1], [2]. Many different ontologies have been developed in various sub-disciplines. For instance, BioPortal [3] currently provides access to more than 500 different biomedical ontologies. Ontologies consist of defined concepts, that are typically structured within trees or acyclic graphs where the concept nodes are interconnected by *is-a*, *part-of* and other semantic relationships. One main application of ontologies is the semantic annotation of different kinds of data objects. For instance, the well-known Gene Ontology (GO) is used to describe molecular functions of genes and proteins [4] and to predict new gene functions [5]. Chemical entities can be described by the Chemical Entities of Biological Interest (ChEBI) ontology [6], and concepts of medical ontologies like SNOMED CT [7] are assigned to documents like electronic health records (EHRs) or case report forms (CRFs). However it is important to note, that in the biomedical domain the term “ontology” is often not used in the sense of formal, axiom-based ontologies but instead for a wide spectrum of simpler terminologies including a.o. thesauri, taxonomies and is-a-hierarchies. The well-known definition of Gruber “*An ontology is an explicit specification of a conceptualization.*” [8] leaves room for variation w.r.t. to the detail of specification [9]. There is a wide spectrum of ontologies of varying expressiveness ranging from simple controlled vocabularies and thesauri to informal and formal “is-a” structures, and, at the highest level of expressiveness, formal ontologies that specify disjoint classes, part-whole relationships and further kinds of logical constraints [9]. The W3C provides a definition for different kinds of non-formal ontologies and calls them knowledge organization systems (KOS). KOS denote a.o. thesauri, classification schemes, subject heading systems and taxonomies and can be expressed by the *Simple Knowledge Organization System* (SKOS) data model [10]. Throughout the paper we will use the term “ontology” for ontologies of varying expressiveness as done by most of the relevant work on biomedical ontology and ontology evolution.

Often there are several ontologies within one domain and they can contain overlapping information. Mappings between such related ontologies interrelate or link corresponding and semantically related concepts and are of high importance for data integration and ontology-based query and analysis tasks. For instance, these mappings support merging several related ontologies into one ontology (e.g. [11], [12]). A prominent huge integrated data source is the Unified Medical Language System (UMLS) [13] built out of more than 100 biomedical ontologies. Moreover, ontology mappings can support a semantic search since ontology-based queries can be enhanced by involving additional ontologies that are interconnected via mappings. Typically, an ontology mapping covers a set of semantic correspondences (links) between the concepts of two different ontologies. The semi-automatic determination of ontology mappings (ontology matching) has been an active research area for more than a decade [14], [15]. Similarly numerous approaches have been proposed to determine biomedical annotation [16], [17], i.e., to link biomedical objects or documents to describing ontology concepts. Such methods produce recommendations that support domain experts in finding correct and complete ontology mappings and annotations.

**Fig. 1:**
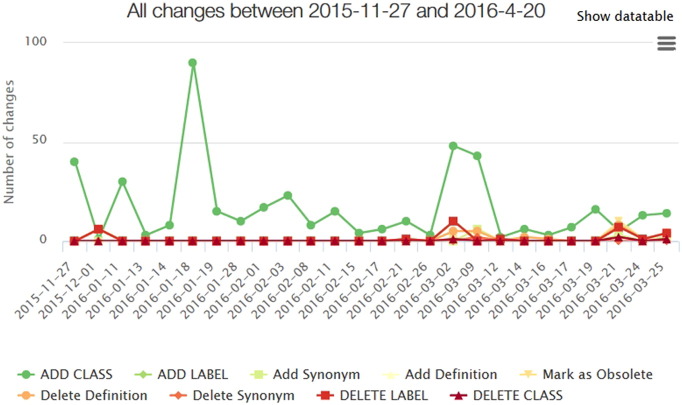
History of changes in Gene Ontology (generated with ontology lookup service [18]).

Usually, ontologies are not static but modified on a regular basis. This process is known as *ontology evolution*. For instance, ontologies need to be changed to incorporate new domain knowledge, remove design errors or to achieve changed requirements. Often ontology development is a collaborative process that is supported by tools such as Protégé [19] or OBO-Edit [20]. In the life sciences, many ontology consortia continuously release new ontology versions. For instance, GO releases a new version every day, while the National Cancer Institute Thesaurus (NCIT) [21] is published on a monthly basis. Fig. 1 exemplarily shows the history of changes between 2015–11 and 2016–04 in GO. Typically, new versions contain improved and extended knowledge such as new concepts (classes), relationships or attributes like synonyms. However, existing knowledge can also be revised or removed, e.g. concepts might be deleted or marked as obsolete. For instance in the shown time period for GO (see Fig. 1), new classes have been added continuously, in 03-2016 some concepts were set to obsolete, and some definitions and class labels have been deleted. To manage the evolution of ontologies it is essential to determine changes, e.g. by analyzing change logs or by computing the difference (Diff) between two given versions of an ontology. Such a Diff is useful to synchronize changes in collaborative ontology development and to adapt dependent applications.

**Fig. 2:**
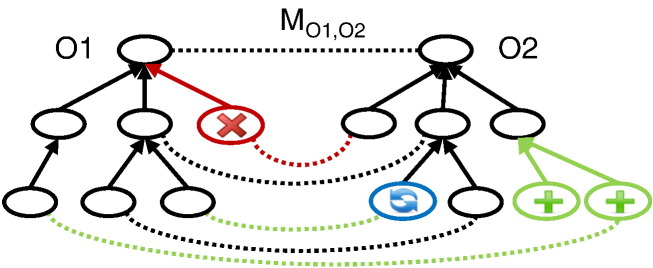
Example ontology and mapping evolution.

The evolution of ontologies has impact on ontology-based applications. For instance, ontology mappings and annotations can become invalid when the underlying ontologies are changed. This is especially critical in highly volatile domains such as the life sciences. Fig. 2 illustrates two ontologies (*O*1 and *O*2) and a mapping between them (*M*_*O*1,*O*2_). In *O*1, one concept has been removed (red) while two concepts have been added to *O*2 (green). Another concept in *O*2 has been revised (blue) e.g., by changing the concept name. These ontology changes have impact on the set of correspondences (dashed lines) and might require changes in the mapping. In the example, one correspondence is associated to a deleted concept, and might therefore be removed. Moreover, the added and revised concept might lead to novel correspondences. Hence, ontology-based mappings can become out-dated as a consequence of ontology evolution. In order to keep mappings up-to-date they need to be migrated to currently valid ontology versions. On the one hand, a manual mapping maintenance can be very time consuming or even infeasible since ontologies and mappings can become very large. On the other hand, automated methods could be simply reapplied on the same data to obtain a valid mapping w.r.t. the current ontology version. However, this can lead to a huge loss in quality since existing mappings might have been manually verified and corrected in the meantime. Just recomputing the results would discard this valuable knowledge. Moreover, usually a smaller part of an ontology is changed such that it seems likely to adapt only affected mapping parts. Therefore, it is useful to apply (semi-) automatic adaptation methods to migrate out-dated ontology-based mapping to currently valid ontology versions.

In this review, we will first introduce the problem of ontology and mapping evolution (Section 2) and then give an overview of recently proposed evolution methods for the biomedical domain and discuss open challenges:

• Methods for ontology evolution have been surveyed in several contexts before (e.g. [22], [23], [24], [25]). Here we will focus on recent approaches that we see relevant for semi-automatic adaptation of ontology-based mappings and applications in the life sciences. This includes novel directions in ontology change detection and prediction and the visualization of ontology evolution. (Section 3)
• We will then discuss requirements for mapping evolution and provide a comparison and overview on existing (semi-) automatic adaptation strategies for ontology-based mappings. (Section 4)
• We will finally outline open challenges and future directions for the evolution of ontologies and ontology-based mappings and applications (Section 5).

## 2 Problem formulation

In this section, we will introduce the basic scenario of ontology and mapping evolution along with an illustrating example. An ontology *O* = (*C*, *A*, *R*) consists of a set of concepts *C* (or classes) that are connected via a set of relationships *R* with different semantics such as *is-a* or *part-of*. Often ontologies form so-called Directed Acyclic Graphs. Each concept is further described by a set of attributes and their associated values *A* such as the name/label, a definition and synonyms. Each concept is uniquely identified by an ID attribute, often called accession number in biomedical ontologies. For one ontology, one or more versions *v* = 1 , … , *n* can be available: *O*^*v*^ = (*C*^*v*^, *A*^*v*^, *R*^*v*^, *t*). Usually, a linear versioning scheme is applied, i.e. each version *O*^*i*^ has a preceding *O*^*i* − 1^ and a succeeding version *O*^*i* + 1^ (except for the first and last version). A version *O*^*v*^ is valid in a specific period of time, namely from the time of release *t* until a new version is released at time *t*^′^ (*t* < *t*^′^). This also holds for all objects (*C*, *A*, *R*) covered by the respective ontology version.

**Fig. 3:**
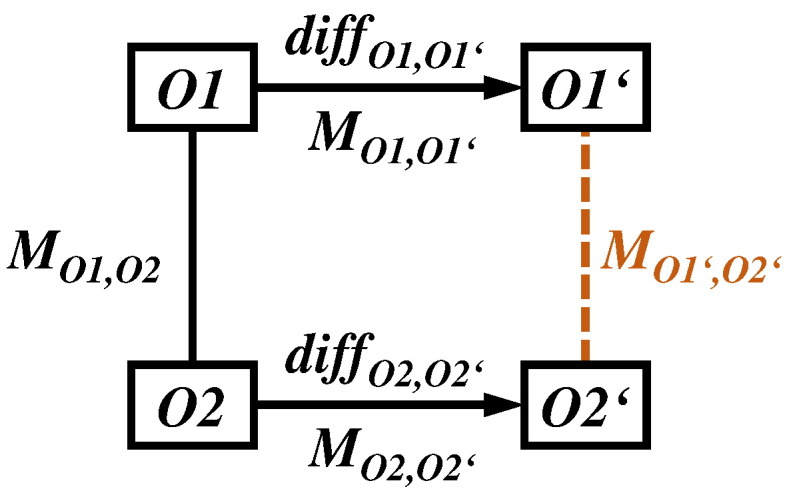
Ontology and mapping evolution scenario.

**Fig. 4:**
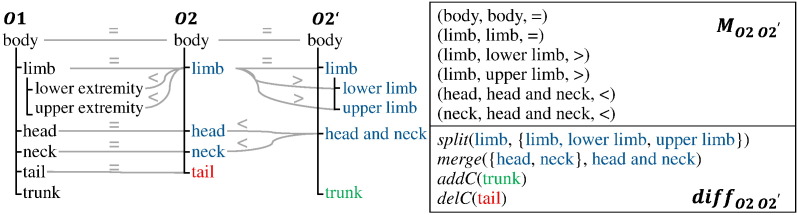
Mapping evolution example.

Fig. 3 shows two ontologies *O*1 and *O*2 each having a succeeding version *O*1′ and *O*2^′^. *O*1 and *O*2 are connected via an ontology mapping *M*_*O*1,*O*2_. Typically, such a mapping consists of a set of correspondences between the concepts of *O*1 and *O*2: *M* = {(*a*, *b*, *sim*, *semType*)| *a* ∈ *O*1, *b* ∈ *O*2, *sim* ∈ [0, 1], *semType* ∈ {=, <, >}}. Beside aligning concepts of different ontologies, it is further useful to identify correspondences between relationship types from different ontologies. Automatic matching techniques usually determine a similarity value *sim* describing the strength of a connection. Correspondences are further described by a specific semantic type (*semType*). Often ontology mappings contain correspondences with equivalence semantics (*equal*, *same-as*, ‘=’). However, also other semantic types can be determined, e.g. *less general* (‘<’) or *more general* (‘>’). For more details on the semantic enrichment of ontology mappings we refer to [26]. In the example in Fig. 4, the mapping between two exemplary anatomy ontologies *O*1 and *O*2 covers five equality and two *less general* correspondences (*lower extremity* and *upper extremity* to *limb*).

When new ontology versions are released, affected mappings and applications should also be adapted to utilize the knowledge of the updated ontology. In the mapping adaptation scenario we are looking for a new mapping version *M*_*O*1^′^,*O*2^′^_ based on new versions of the ontologies (see Fig. 3). Note that an ontology mapping might also be only affected by changes in one of the ontologies. Instead of recomputing the mapping from scratch, it is desirable to reuse the previous mapping *M*_*O*1,*O*2_ as much as possible. Therefore, adaptation methods should make use of an evolution mapping between *O*1 and *O*1^′^ as well as *O*2 and *O*2^′^. This evolution mapping can be an ontology mapping covering a set of semantic correspondences between the old and new versions (e.g. see *M*_*O*2,*O*2^′^_ in Fig. 4). Alternatively one can determine a diff evolution mapping covering a set of changes. For instance, in the example *diff*_*O*2,*O*2^′^_ covers a set of change operations such as a split of the concept *limb* into *limb*, *lower limb* and *upper limb*, a merge of *head* and *neck* into *head and neck*, as well as the concept deletion *delC*(tail) and addition *addC*(trunk).

Beside ontology mappings there are other *ontology-based mappings* such as annotation mappings or ontology-based queries that are affected by ontology evolution. Annotation mappings consist of a set of correspondences between biomedical objects and an ontology to describe the association or description of these objects by ontology concepts. Ontology-based queries use ontology concepts to semantically query data and are thus also affected by changes in the ontology. While we focus on the adaptation of ontology mappings in this article, in Section 4 we will also include one related adaptation approach for ontology-based annotation mappings.

## 3 Ontology evolution

The life sciences are a highly dynamic domain by nature. New findings lead to a constant renewal of domain knowledge making it richer over time. However, this evolution deeply impacts domain ontologies, forcing experts to regularly revise their content. Ontology evolution is therefore a research field that has gained more and more interest over the past years through a joint effort of the biomedical and Semantic Web communities. Since this subject has been recently surveyed [22], [24], [25], we focus on pointing out some interesting novel investigations, that are or will be particularly important to improve the adaptation process for ontology-based mappings and applications. This covers ontology change detection, the visualization of ontology evolution and ontology change prediction and tracking. Further challenges will be discussed in Section 5.

### **Change detection**

**Fig. 5:**
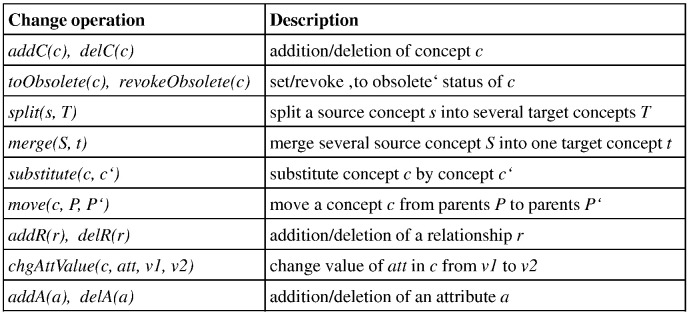
Change operations in COnto-Diff [27], [28].

First of all, biomedical ontologies are much bigger than those of other domains so it is hard to see changes between ontology versions at a glance. Moreover, since there is no standard language for documenting changes occurring in ontologies, *Diff* computation approaches are especially important to identify changes between different ontology versions. PromptDiff was the first relevant initiative able to identify the differences between two ontology versions [29]. More recently, COnto-Diff [27] offered the user a way to specify change patterns and dedicated rules to determine a more compact and semantically more expressive diff representation. The compact diff representation covers complex ontology changes such as merging, splitting and moving of concepts or the addition and deletion of large sub-graphs. Fig. 5 shows some important change types that are detected by COnto-Diff. Recently, Yingjie et al. [30] introduced a method to detect conflicts between several sequences of ontology changes. For instance, such conflicts can occur during collaborative ontology evolution where different stakeholders and viewpoints are usually involved. The study combines change detection and inconsistency checking methods in order to identify conflicting change sequences in ontology evolution. Moreover, Dos Reis et al. defined lexical and semantic change patterns based on the evolution of several medical ontologies [31]. These change patterns allow to characterize the way attribute values of concepts evolve, e.g., if the observed changes are likely to modify the meaning of an attribute value. The change operations and change patterns as determined by diff algorithms and other methods are very useful to maintain ontology-based mappings and other dependent applications.

The need for a retrospective identification of differences between versions can be avoided during editing when ontology changes are well-documented including reasons of changes. For instance, it is important to document change operations in an upper level formal ontology such as Basic Formal Ontology (BFO) in order to allow for an appropriate change propagation into dependent domain ontologies [32]. Evolutionary terminology auditing (ETA) allows for measuring the quality improvements of formal ontologies and different kinds of terminologies over successive versions, and requires that ontology editors keep track of changes and their motivation for the respective changes (e.g., [33], [34]). However, so far there is no standard language for documenting ontology evolution.

### **Visualizing ontology evolution**

It has become more and more important to provide intuitive ways of visualizing ontology evolution (e.g., [18], [35], [36], [37], [38], [39]). For users it is particularly important to understand the evolution of ontologies they use in order to be able to assess possible influences on their ontology-based applications. For instance, quite recently a new version of the widely used ontology lookup service^1^[18] was introduced to inform users about ontology change histories. The CODEX^2^[35] tool allows users to explore complex changes computed by COnto-Diff. WebProtégé^3^[36] supports the tracking of ontology changes and provides precisely defined, OWL-related ontology changes and change lists. Diff Abstraction Networks [37] were introduced to summarize, visualize and highlight ontology changes. It further seems intuitive to provide a dynamic graph visualization perspective for time-varying ontologies [40]. For instance, the tool REX^4^[38] gives an aggregated view on differently evolving ontology regions and allows users to navigate from the root into stable or strongly evolving ontology regions using a fish-eye zoom. However, still much work needs to be done to improve ontology evolution visualization techniques allowing for compact as well as detailed views e.g. on precisely defined changes of axioms in formal ontologies.

### **Ontology change prediction**

In the last years, the tracking and prediction of ontology evolution has gained attention. This is of special interest for collaborative ontology editing and development as well as for the migration of ontology-based applications. Also change prediction methods can not guarantee to be perfectly correct and precise, they can support users in planing and managing adaptation processes, e.g. by precociously indicating possibly impacted parts of dependent mappings and applications. Current relevant work includes [41] where the authors focused on tracking the collaborative processes behind the evolution of an ontology, i.e., the changes made by contributors over time. Wang et al. investigate the way ontology editors behave when they modify an ontology and predict future modifications [42]. Moreover, Pesquita & Couto used machine learning techniques to predict which branch of the Gene Ontology is likely to expand in the future release using supervised learning methods [43]. Tsatsaronis et al. implement temporal classifiers to predict future extension of the MeSH controlled terminology using MeSH-indexed PubMed articles [44].

## 4 Adaptation of ontology-based mappings

One of the additional challenges of ontology evolution is to keep dependent artifacts such as ontology-based mappings up-to-date. Several evolution studies in the life science domain (e.g., [45], [46], [47]) showed frequent and continuous changes for both, the considered ontologies and ontology-based mappings. In particular, the results in [46] showed significant instabilities for mappings created by automatic ontology matching techniques, e.g., utilizing the similarity of concept names and their synonyms for deriving correspondences. These observations underline the importance of (semi-) automatic adaptation strategies that can reuse and extend previous mappings instead of completely recomputing the mappings when an ontology changes.

In the following we will first discuss requirements for the adaptation of ontology-based mappings. In Section 4.2 we will then discuss adaptation strategies for ontology-based mappings in the context of ontology evolution and compare them based on the introduced requirements. Approaches for the more general problem of mapping maintenance and repair are discussed in [48].

### 4.1 Requirements

(Semi-) automatic mapping adaptation strategies need to achieve several requirements to be useful for applications and users:

• **Mapping quality:** Mapping adaptation methods need to determine high-quality mappings. The correspondences in migrated mappings need to be correct and complete, i.e., methods need to achieve high precision and recall values.
• **Mapping validity:** An adapted mapping needs to cover solely correspondences to valid concepts from the new ontology versions. Mappings must not contain any inconsistent correspondences, e.g., to obsolete or deleted concepts.
• **Inclusion of added concepts:** Mapping adaptation methods need to involve ontology extensions such as concept additions in order to obtain a complete result mapping. This is especially relevant for highly volatile domains such as the life sciences where ontologies are heavily extended.
• **Reduction of manual effort and user involvement:** The adaptation process should be largely automatic to limit the manual effort, especially for very large ontologies and mappings. One main aim is to reuse large parts of an existing mapping and avoid a full re-determination. User involvement is very important, but should mainly be restricted to verify and potentially revise automatically updated mappings.
• **Scalability and efficiency:** Mapping adaptation approaches should be efficient and scalable to process large ontologies and mappings as common in the biomedical domain.
• **Support for semantic mappings:** Adaptation methods need to consider the actual semantics of correspondences. Beside *equality* relationships ontology mappings can cover further semantic correspondences such as *less/more general* or *part-of/has-a*. Therefore, sophisticated methods are necessary to correctly determine the semantic type of a correspondence during the migration process.

### 4.2 Approaches

We will now discuss existing adaptation approaches for ontology-based mappings that are affected by ontology evolution. Four approaches have been explicitly proposed to adapt ontology mappings. One further approach is highly related since it deals with the adaptation of ontology-based annotations as a consequence of ontology evolution. The different approaches are summarized in Table 1 w.r.t. the posed requirements. In the following, we first introduce the main idea for each approach and then comparatively discuss the approaches.

The first approach to automatically evolve or adapt ontology mappings has been proposed by Martins and Silva [49]. Their aim is to resolve possible mapping inconsistencies depending on the previously applied ontology evolution strategy. The authors distinguish between elementary changes in ontology mappings such as additions and deletions of attribute values in source or target concepts, as well as composite changes like updates of attribute values. The mapping evolution process tries to identify the previously applied ontology evolution process for every affected correspondence. The authors discuss a user-driven and a semantic mapping evolution process. In particular, they discuss one mapping evolution strategy in case of concept deletions in detail, but do not focus on other change types. It remains unclear if all possibly invalid correspondences will be adapted by their approach. The evaluation uses small exemplary ontologies of 15–25 concepts and does not consider the quality of the adapted mappings.

Hartung et al. [39] developed the web tool OnEX that also supports the adaptation of biomedical annotations. The system first computes basic change operations between the old and new ontology version. According to the type of ontology change the system proposes one or more possibilities to adapt an affected annotation. OnEX provides basic mapping adaptation strategies for information-reducing change operations such as concept deletion, setting concepts to obsolete or concept fusion but not for information-extending operations like concept additions. The approach can be applied for several predetermined life science ontologies but has not been specified formally and was not evaluated.

Khattak et al. [50], [51] present an automatic adaptation approach relying on a partial re-computation of ontology mappings that are affected by ontology evolution. The approach uses a *Change History Log* (CHL) [52] to detect ontology changes such as *create*, *update*, *delete* for concepts and attributes. Changed elements in the source or target ontology of a mapping are automatically matched with the complete current version of the other ontology. The approach only reuses the completely unaffected part of a mapping, discards all affected correspondences (independent of the change type) and adds all newly computed correspondences (output of the matching step). In the evaluation, mappings between different life science ontologies such as Adult Mouse Anatomy Ontology (MA) and NCIT are automatically generated by different match tools (e.g., Falcon [53], TaxoMap [54]). Then 25 ontology changes (mainly additions) are induced manually, i.e., it does not rely on real ontology versions. The studies show an improvement w.r.t. execution times compared to the complete mapping re-computation but does not evaluate the quality of the produced mappings.

Groß et al. [28]^5^ present two approaches for adapting ontology mappings. The composition-based and diff-based adaptation approaches both rely on the reuse of existing mappings (e.g. *M*_*O*1,*O*2_ in Fig. 3) as well as the use of evolution mappings (e.g. between *O*1 and *O*1' in Fig. 3). The first approach uses a composition of the old ontology mapping with an evolution mapping containing the semantic correspondences between the old and new ontology version. Mapping composition makes use of the transitivity criterion where two correspondences (*a*, *b*, =) (*a* ∈ *O*1 , *b* ∈ *O*2) and (*b*, *c*, =) (*b* ∈ *O*2 , *c* ∈ *O*2^′^) are combined to a new correspondence (*a*, *c*, =). The authors propose a set of rules to achieve the correct semantic type for the migrated correspondence, e.g. two equality correspondences can be combined to one equality correspondence. Complex cases like the combination of one less general (*a*, *b*, <) with one more general correspondence (*b*, *c*, >) cannot be resolved automatically. In these cases the user can be involved to decide for the correct type. The second approach makes use of a diff evolution mapping covering individual ontology changes computed by COnto-Diff [27] as well as a set of change handlers to migrate affected correspondences according to the change type. The approach applies the same semantic type rules as the composition-based approach. The diff-based approach can handle basic changes like attribute value changes as well as complex change types such as concept splits or merges. The evaluation analyzes the quality of adapted mappings between three very large life science ontologies (NCIT, SNOMED CT, Foundational Model of Anatomy) and could show a very high effectiveness, in particular for the diff-based approach, with F-Measure values between 90% and 94%.

Dos Reis et al. [55]^5^ propose a similar approach than the diff-based scheme of [28] using so-called mapping adaptation actions (MAAs) to keep mappings up-to-date for different ontology changes. Ontology changes are computed using the COnto-Diff algorithm [27] and further categorized into revision, deletion and addition of ontology elements (*C*, *A*, *R*). Moreover, the authors distinguish between different mapping changes (remove, addition, move, derivation, modification) and propose one MAA for each mapping change type. For instance, the derivation is a composed action where an existing correspondence is reused as a modified copy of this existing correspondence. The modification action supports the adaptation of mappings with different types of semantic relations instead of only considering equivalence correspondences. The evaluation analyzes ontology and mapping changes for three large life science ontologies and existing mapping versions between them (NCIT, SNOMED CT, ICD-9-CM). The evaluation does not assess the quality by computing F-Measure values for migrated mappings, but instead identifies the effectiveness of the approach by computing proportions of the proposed MAAs as an actual consequence of different ontology change types. These results vary depending on the types of ontology and mapping changes, e.g. 65% of the *toObsolete* operations lead to a correspondence adaptation by replacing the obsolete source concept with its super concept.

#### 4.2.1 Discussion

Table 1 summarizes the discussed approaches for different criteria addressing the introduced requirements. Most approaches produce valid mappings w.r.t. the new ontology version(s). To detect changes between ontology versions, two of the approaches [28], [55] use a complex diff evolution mapping covering semantically meaningful change operations such as *split* or *merge*. Three approaches rely on a *basic diff* evolution mapping that covers the basic change operations *add*, *delete* and *update* for concepts, relationships and attributes. The two adaptation approaches [28], [55] are most advanced as they also consider not only complex changes but also new ontology concepts to find additional correspondences and they support semantic mappings with both equality and more/less general relations. The importance of supporting non-equality relationships in ontology mappings is confirmed in a further study [56] for mappings between SNOMED CT and ICD, since SNOMED CT tends to cover additional and more detailed knowledge. Overall three approaches [28], [51], [55] generate new correspondences by applying standard ontology matching techniques to align added concepts from one of the ontologies with the respective other ontology.

The evaluations of the approaches showed to be quite heterogeneous and difficult to compare, e.g. they differ in the used ontologies and analysis focus. While several studies considered large ontologies, efficiency and scalability have not yet been analyzed in detail. Only two studies evaluated the quality of the adapted mappings. Dos Reis et al. [55] analyzed how often their proposed adaptation actions actually occurred in real world ontology mapping versions. Groß et al. [28] evaluated the mapping correctness and completeness by computing precision and recall (F-Measure of 90–94%) for the automatically adapted mappings compared to the actually released mapping versions. For sure, users cannot rely on fully automatically generated mappings. Therefore, the produced recommendations need to be verified and corrected by expert users. Some approaches require and allow for user interaction, e.g., by marking uncertain correspondences for verification [28]. However, no system provides a really comfortable way such as a visualized workflow to guide expert users through the verification and quality control process.

Overall, there are promising strategies to semi-automatically migrate ontology-based mappings when the underlying ontologies evolve. However, further research and careful evaluation for different kinds of ontologies and ontology-based applications are still necessary as well as the integration of the approaches within user-friendly tools.

## 5 Open challenges and future directions

We see several important directions for future work on ontology and mapping evolution.

### Evolution of semantic mappings

There are only few systems that can handle and generate different semantics of ontology-based mappings, especially in the context of ontology evolution. Some approaches already focus on generating semantically enriched mappings between different ontologies by identifying, e.g. *is-a* or *part-of* correspondences beside the typical equality relationships (e.g. [26], [57]). Considering the evolution of ontologies, none of the existing change detection approaches actually finds semantically enriched evolution mappings between different ontology versions. For instance, there might be different semantics for merge operations such as a *part-of* or an *is-a* merge of several concepts. Such semantically enriched evolution mappings could then be used to correctly adapt ontology mappings and ontology-based annotations. Moreover, novel migration approaches need to pay special attention to ontology-based annotation mappings with typically domain-specific semantics (e.g. *is involved in*, *has function*).

### Evolution of formal ontologies and mappings

Current mapping adaption approaches rely on change operations that have been determined using diff algorithms covering changes in the ontology structure, concept attribute changes and many others. However, existing methods need to be extended to also involve change operations between different versions of formal axiom-based ontologies. For instance, in [32] change operations in an upper level ontology are formally documented to allow the propagation of changes into domain ontologies that rely on the upper level ontology and therefore need to be adapted. Similar methods will be needed to also adapt mappings between formal ontologies. Current approaches will be useful to a certain degree, but need to be extended to include changes on axiom-based expressions as well as thorough verification based on formal reasoning methods.

### Prediction methods and verification of recommendations

The mapping adaptation and verification process can benefit from novel developments on ontology evolution such as discussed in Section 3. Algorithms that aim at the prediction of ontology changes based on the history of ontologies can also be used or extended to identify annotations or correspondences in ontology mappings that are likely to undergo evolution in the near future. Ontology and mapping curators could thus be supported by highlighting dynamic ontology parts in order to focus the revision task on the respective ontology and mapping parts. Current mapping adaptation approaches produce recommendations how to migrate a mapping, however expert users are not well supported in correcting these results. In order to improve the verification and quality control process, it is important to develop systems that combine ontology evolution analysis, change prediction and recommendation generation to migrate existing ontology-based mappings and other applications. This process can also benefit from current developments and insights on user involvement in ontology matching tasks [58]. Novel systems should include intuitive and practical visualization solutions that guide human experts by pointing to invalid mappings and adaptation recommendations.

### Evolution of merged ontologies

The existing approaches only consider the migration of ontology-based mappings between two different sources. However, there are more complex scenarios where a holistic view with more than two sources is needed. For instance, there are efforts to merge several biomedical ontologies in order to provide one integrated ontology in a domain of interest. Beside the huge UMLS there are other merged ontologies such as Uberon [59] in the anatomical domain. Such merge processes are currently done largely manually and thus tedious and error-prone. Furthermore, they cannot easily deal with the continuous changes of the underlying source ontologies. A more automated approach would be to utilize mappings between the source ontologies and merged target ontology and apply a mapping-based merge algorithm [11]. For new versions of the source ontologies, the ontology mappings and merge result need to be adapted. Such an evolution-aware merge approach is challenging and involves several interrelated subproblems.

1. The source ontologies evolve based on their requirements to incorporate new knowledge and follow their design guidelines.
2. This has impact on the mappings between the source ontologies (e.g. SNOMED CT, NCIT) and the integrated target ontology (e.g. UMLS), i.e. those mappings need to be adapted accordingly.
3. The integrated ontology follows its own design guidelines that can result into changes independent from the source ontologies.
4. The integrated ontology needs to be adapted accordingly by taking the source ontology and mapping changes as well as its own changes into account. During this process existing curated knowledge must not disappear, e.g., a change in the integrated ontology should not be overwritten during an update process. However, the evolution in the source ontologies should still be reflected appropriately in a merged ontology.

The described scenario shows that those inter-dependencies between several sources as well as changes in the individual sources require more advanced data and ontology migration approaches.

### Evolution of multilingual ontologies and mappings

Ontology and mapping evolution also need to deal with the maintenance of multilingual ontology mappings. Contrary to other domains, many ontologies in the life sciences are widely accepted as standards to encode data in different countries and to facilitate international data exchange among health professionals. For instance, this is the case for the International Classification of Diseases (ICD) to encode diagnoses in different countries.^6^ To support international usage, ontologies have been made available in a wide variety of natural languages [60] and there are ongoing efforts to provide translations for mono-lingual ontologies as highlighted by the road-map to obtain a multilingual BioPortal [61]. This requires to properly evolve several multilingual ontology versions and the associated cross-lingual ontology mappings [62] or ontology translation mappings [61] according to changes in the reference (“original”) ontology. Basically one can distinguish two cases [61]:

1. A multilingual ontology covers different natural language representations using several concept labels.
2. Two mono- (or multi-) lingual ontology representations are interconnected by a translation mapping.

For case (1) a concept label change in one language has to trigger label changes for other language representations of this label. For case (2) changes in the structure of one or both of the ontologies need to trigger adaptations in the multilingual translation mapping and possibly in the respective other ontology. The multilingual ontology mapping task differs from the adaptation of merged ontologies, where changes in several source ontologies need to be migrated into one merged version.

## 7 Conclusion

In this survey, we outlined recent advances on ontology and mapping evolution in the biomedical domain. While there has been considerable work in the domain of ontology evolution, more work is still necessary to deal with ontology changes in applications that rely on those ontologies. We discussed novel directions on ontology evolution, and presented an overview on existing adaptation approaches for ontology-based applications that are affected by ontology evolution. Finally, we outlined open challenges and interesting future directions. In particular, we see the need for more research for a correct maintenance of merged ontologies, multilingual ontologies and the involved ontology mappings.

## Figures and Tables

**Table 1:** Adaptation approaches for ontology-based mappings.

	Martins and Silva 2009 [49]	Hartung et al. 2009 [39]	Khattak et al. 2012, 2015 [50], [51]	Groß et al. 2013 [28]	Dos Reis et al. 2013 [55]
*Description*	Application of ontology evolution strategy	Migration via GUI for pre-defined ontologies	Re-computation for changed ontology parts	Composition- and diff-based adaptation	Adaptation via mapping change actions

*Input*					
Outdated/adapted mapping	Ontology mapping	Ontology-based annotations	Ontology mapping	Ontology mapping	Ontology mapping
Evolution mapping	Simple diff	Simple diff	Simple diff	Ontology mapping or complex diff	Complex diff

*Mapping validity*	?	Yes	Yes	Yes	Yes

*Use of added concepts*	No	No	Yes	Yes	Yes

*User interaction*	(Semi-) automatic	(Semi-) automatic	Automatic	(Semi-) automatic	Automatic

*Semantic mappings*	Equivalence	–	Equivalence	Equivalence, more/less general	Equivalence, more/less general


*Evaluation*					
Ontology size (| concepts |)	15–20	≤ 97.000	≤ 42.000	≤ 319.000	≤ 396.000
Ontology evolution	Manual changes	Ontology versions	Manual changes	Ontology versions	Ontology versions
Quality	No	No	No	Yes (precision, recall)	Partial (relevance of adaptation)

## Footnotes

1 http://www.ebi.ac.uk/ols.

2 www.izbi.de/codex.

3 http://webprotege.stanford.edu.

4 www.izbi.de/rex.

5 Previous work of the authors.

6 http://www.epsos.eu.
